# A cost-effectiveness analysis of baloxavir marboxil versus oseltamivir for seasonal influenza treatment in China

**DOI:** 10.3389/fphar.2025.1697645

**Published:** 2026-01-02

**Authors:** Fangyu Zheng, Yuekun Tang, Hongli Jiang

**Affiliations:** 1 School of Public Health, Fudan University, Shanghai, China; 2 Eye & Ent Hospital of Fudan University, Shanghai, China

**Keywords:** cost-effectiveness, seasonal influenza, baloxavir marboxil, antiviral agents, decision tree

## Abstract

**Objectives:**

In this study, we aimed to evaluate the cost-effectiveness of baloxavir marboxil versus oseltamivir for treating seasonal influenza among both high-risk and otherwise healthy populations in China from the healthcare system perspective.

**Methods:**

A decision tree model was constructed to evaluate the cost-effectiveness of two drugs. This model was integrated with a transmission dynamics model to assess the impact on controlling influenza spread. Model parameters for epidemiology, costs, and utilities were derived from real-world data on treatment costs from secondary and tertiary hospitals across China, the phase III clinical trial of baloxavir (CAPSTONE), and published literature.

**Results:**

Baloxavir was found to be a dominant strategy compared to oseltamivir in both populations, yielding lower costs and higher QALYs. For the high-risk population, baloxavir treatment would save $2.5468 per patient and gain an additional 0.00018 QALYs. In addition, for the otherwise healthy population, it would save $1.1278 per patient and gain an additional 0.00024 QALYs. One-way sensitivity analysis identified QALYs for influenza and hospitalization costs as the most influential parameters for the high-risk population, whereas the number of infections, ICU fatality rate, and hospitalization costs were key drivers for the otherwise healthy population. Probabilistic sensitivity analysis confirmed the robustness of these findings.

**Conclusion:**

Baloxavir is a cost-effective alternative to oseltamivir for seasonal influenza treatment in China. It can improve patient outcomes and reduce influenza transmission at a societal level, offering value to the healthcare system.

## 1 Introduction

Influenza is an acute respiratory infection caused by the influenza virus, known to cause severe upper respiratory tract infections that contribute to high morbidity and mortality worldwide (Yi et al., 2023). According to the World Health Organization (WHO), influenza annually results in 3–5 million cases of severe illness and 290,000 to 650,000 respiratory disease-related deaths globally (Li et al., 2023). National influenza surveillance data from China from 2010 to 2020 estimated that the cumulative numbers of influenza infections, morbidity, and medical visits were 89.453 million, 59.216 million, and 38.415 million, respectively (Wang et al., 2022). Notably, the elderly population faces a higher risk of severe illness and death from influenza. Studies in China have indicated that influenza-related excess mortality is substantially higher among individuals older than 65 years than in those aged 0–64 years, with 80%–95% of influenza-related excess mortality occurring in individuals aged 65 years and older (Li et al., 2019; Wang et al., 2014).

Influenza can be managed using antiviral drugs, which can effectively shorten the duration of illness and prevent severe complications (Igarashi et al., 2025; Liu et al., 2021). Current antiviral treatments are dominated primarily by neuraminidase inhibitors. Oseltamivir is the most prominent of these, holding a global market share of 47.5% in 2024 and accounting for 76.3% of outpatient prescriptions. Over 220 million units have been distributed worldwide. Zanamivir, administered via a dry powder inhaler, holds a 21.2% share of the anti-influenza drug market. Prescriptions in the Asia–Pacific region grew by 12.8% due to updated delivery devices. However, limitations in the inhalation route led to a 9.3% decrease in its use among elderly patients. For critically ill patients who are resistant to oseltamivir and for inpatients who are unable to tolerate oral medications, intravenous peramivir remains a critical treatment option. In 2024, it held a 13.8% market share, with Japan accounting for 49.1% of global usage as its largest consumer (Market Growth Reports, 2025). Notwithstanding their widespread clinical use, neuraminidase inhibitors are associated with several recognized limitations. These include dependence on viral subtypes for efficacy, the emergence of drug resistance (such as the NA-H274Y mutation against oseltamivir), strict dependence on early administration, and reduced effectiveness against strains such as H7N9 (Jones et al., 2023; Lampejo, 2020). Although global surveillance indicates relatively low resistance rates to neuraminidase inhibitors (between 0.5% and 0.6% from 2018 to 2020), this does not negate the risks posed by viral evolution and subtype variation. Overall, these limitations highlight the need for antiviral drugs with different modes of action (Lai et al., 2025).

Baloxavir marboxil (hereinafter baloxavir) is a new drug with anti-influenza virus activity. Its mechanism of action differs from that of other existing antiviral therapies; it targets the cap-dependent endonuclease of influenza A and B viruses, inhibiting the cap-snatching step in viral mRNA transcription and thus suppressing viral replication (Omoto et al., 2018). It is an orally administered, small-molecule antiviral drug for the treatment of influenza (Cai et al., 2024), which is currently available in two formulations: a tablet and a dry powder suspension. The tablet is indicated for previously healthy children aged ≥5 years and adults, along with adolescents aged ≥12 years and adults at high risk for influenza-related complications. Dry powder suspension is primarily indicated for children aged 5–12 years. The recommended dose for all formulations is based on body weight: individuals weighing between 20 kg and 80 kg are recommended to take 40 mg, whereas individuals weighing 80 kg or more are recommended to take 80 mg. For children weighing less than 20 kg, the recommended dose of the dry suspension is 2 mg/kg (National Drug Information Query System, 2021). Regarding safety, common adverse reactions to baloxavir include diarrhea and infectious diseases, such as bronchitis. Post-marketing surveillance has also reported psychiatric events, including delirium and behavioral abnormalities, along with allergic reactions and severe skin reactions (Lai et al., 2025). Despite the reported adverse reactions, post-marketing data from studies involving over 3,000 patients indicate an overall favorable safety profile. In particular, only approximately 11.15% of patients reported adverse reactions within 7 days of administration, all of which were mild to moderate in severity. The majority of patients recovered within 3 days (Nakazawa et al., 2020). Additionally, baloxavir can be therapeutic with a single dose of the drug (Sato, 2025) and shows comparable clinical efficacy to oseltamivir, which is administered twice daily for 5 days (Ison et al., 2020). Studies in several countries and regions have shown that baloxavir significantly reduces the duration of influenza symptoms and shortens the time to symptom relief and fever reduction compared to placebo and oseltamivir (Hayden et al., 2022; Ikematsu et al., 2024; Goto et al., 2024). In addition, a Japanese study found that compared to oseltamivir, baloxavir reduced hospitalization rates, pneumonia, and the use of additional antiviral medications in influenza B patients. Moreover, according to a U.S. study, home transmission (17.8% vs. 26.5%) and emergency room visits (0% vs. 4.6%) were also lower in the baloxavir group than in the oseltamivir group (Takazono et al., 2024; Best et al., 2024). In summary, baloxavir may be able to reduce symptom duration, influenza transmission, and healthcare resource use, and may be a more beneficial treatment modality than oseltamivir.

In February 2018, baloxavir received its first global approval in Japan (Heo, 2018) and was launched in China in April 2021 (Administration, 2021). Despite its clinical benefits, economic evaluations of baloxavir have been limited; so far, only a few studies have examined the cost-effectiveness of baloxavir, including a Japanese study in a high-risk population and a healthy population, both of which demonstrated that the incremental cost-effectiveness ratio (ICER) of baloxavir was below the willingness-to-pay threshold (¥5 million/QALY gained) compared to laninamivir (Skrzeczek et al., 2021; Dronova et al., 2021). In addition, studies in the United States and the United Kingdom agreed that baloxavir was cost-effective compared to oseltamivir (Kommandantvold et al., 2024a; Kommandantvold et al., 2024b). In the Netherlands, it was demonstrated that baloxavir was a cost-effective treatment option for seasonal influenza, but as antiviral drugs were not currently recommended in the Netherlands, the control regimen was symptomatic treatment, primarily with paracetamol, rather than oseltamivir (van der Pol et al., 2024). However, due to differences in prevalence and treatment practices, findings from studies in other countries do not necessarily hold true in China, so in July 2024, a research team led by Yawen Jiang filled a gap in the economic evaluation study of baloxavir in China (Jiang et al., 2024). Nonetheless, the study’s cost data were based on previous observational studies and may not fully reflect the actual visits for influenza in Chinese patients. Therefore, we intend to further explore the economics of baloxavir on this basis by using real-world data.

Before baloxavir was launched in China, oseltamivir was one of the most commonly used antiviral treatments for influenza. In this study, we aimed to use real-world data from patients with influenza-like illness in China to estimate the cost-effectiveness of baloxavir *versus* oseltamivir in both the high-risk and otherwise healthy populations.

## 2 Methods and data

### 2.1 Model overview

**FIGURE 1:**
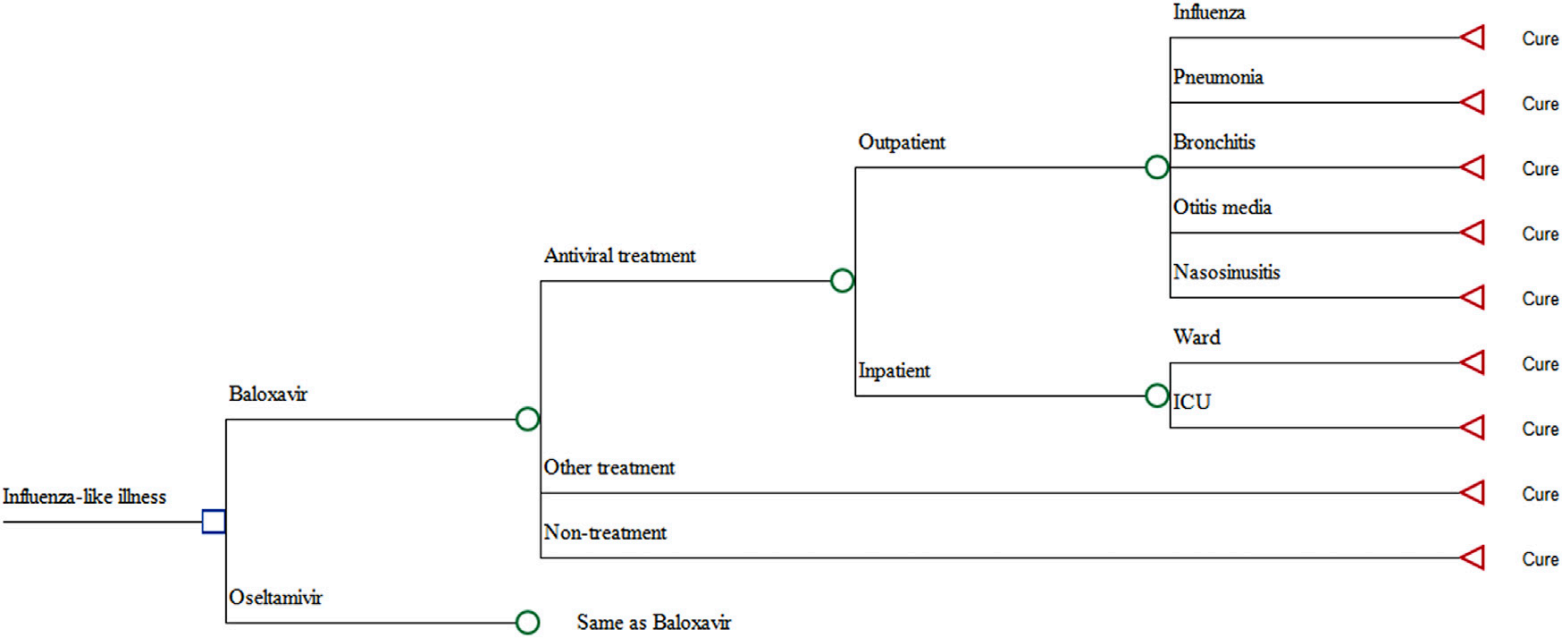
Model structure.

A decision tree model, developed from the healthcare system perspective, was utilized to evaluate the costs and QALYs of baloxavir and oseltamivir throughout one influenza season (referred to as 1 year below). The structure of the decision tree is shown in Figure 1. In addition, the following events were considered: the number of people infected by the treatment regimens of baloxavir and oseltamivir for cases with influenza-like illness, the three subsequent treatment regimens for the transmitted population, and healthcare utilization. The number of people infected was derived from the predicted results of the transmission dynamics model (Jiang et al., 2022), the epidemiological data were derived from the phase III clinical trial (Shionogi, 2018a; Shionogi, 2018b), and cost data were derived from real-world data in China (SuValue ®).

The phase III clinical trial (CAPSTONE) (Shionogi, 2018a; Shionogi, 2018b) was a randomized, double-blind study conducted in a global multicenter to evaluate the efficacy and safety of baloxavir for the treatment of influenza. The population included high-risk individuals (defined in more detail below) aged 12 years and older, and the otherwise healthy population.

The real-world data used in this study were sourced from SuValue’s database, which collected de-identified medical records from 111 secondary and tertiary hospitals across 17 provinces and directly controlled municipalities in China during 2020. The data were utilized to analyze information on complications, treatment costs, and illness duration among patients with seasonal influenza. The exclusion criteria were as follows: in China, influenza diagnosis is usually less dependent on laboratory tests and more dependent on clinical experience. Therefore, we included cases of influenza-like illness in this study and excluded patients presenting with multiple comorbidities at a single visit, according to the assumptions outlined in the following subsection.

### 2.2 Assumptions

The research context was particularly shaped by the prevailing medical practices in China, where physicians frequently adopt empirical treatment strategies for influenza without conducting laboratory tests to confirm the diagnosis. Consequently, the types of complications assessed in this study reflect real-world scenarios and data derived from phase III clinical trials of baloxavir. The complications considered for outpatients included pneumonia, bronchitis, sinusitis, and otitis media. Given that hospitalization costs were similar across different complications, inpatients were categorized into general wards and intensive care units (ICUs) without differentiating the costs associated with each type of complication.

Regarding the willingness-to-pay (WTP) in China, Dan Cai et al. derived a weighted average from several studies and pooled data on population mortality, health utility, and age distribution, suggesting a WTP close to 1.5 times gross domestic product (GDP) *per capita* (Cai et al., 2022). Moreover, Ziping Ye et al. reported a sample average WTP of 1.7 times the GDP *per capita* from a survey (Ye et al., 2022). Despite the lack of an official cost-effectiveness threshold in China, our study adopted the conventional approach of using three times the *per capita* GDP of China in 2022 as the threshold, amounting to $38,223, converted using the average annual exchange rate of 6.73 CNY/USD for 2022 (National Bureau of Statistics of China, 2022).

To simplify the model, we made the following assumptions:

1. Each patient may contract seasonal influenza only once a year. After an influenza virus infection, the body develops a certain level of immunity that usually prevents re-infection with the same strain of the virus in the short term (Jiang et al., 2022).
2. A maximum of one complication was assigned to each patient. In the transmission dynamics model of influenza, a single major complication is usually assumed for each patient; because this study incorporates results from such a model, this assumption is consistent with the underlying transmission dynamics model (Jiang et al., 2022).
3. Based on the results of the phase III clinical trial, it was assumed that patient deaths occurred only in the ICU (Shionogi, 2018a; Shionogi, 2018b).

### 2.3 Study population

The study population was divided into otherwise healthy and high-risk individuals, both aged >12 years. The criteria for defining the high-risk population were aligned with those used in the CAPSTONE study, the phase III clinical trial mentioned in the previous section, which included at least one of the following criteria: 1) older than 65 years; 2) body mass index (BMI) ≥ 40 kg/m2; 3) presence of asthma or chronic lung disease, endocrine disorders, or residence in a long-term care facility; or 4) presence of compromised immune function, neurological or neurodevelopmental disorders, heart disease, blood disorders, and metabolic disorders.

### 2.4 Parameter inputs

#### 2.4.1 Clinical data

Based on the consistency of the model parameters, which were referenced from the transmission dynamics model, it was assumed that 10% of influenza patients received antiviral treatment and another 29% of influenza patients received other treatment, that is, simple influenza only, and that the cost of their treatment was taken as the portion of influenza-related medical costs that did not include the cost of antiviral medications (Jiang et al., 2022).

The mortality and complication incidence rates for seasonal influenza treated with baloxavir and oseltamivir were derived from phase III clinical trials (Shionogi, 2018a; Shionogi, 2018b). Notably, no hospitalizations were recorded for baloxavir in the otherwise healthy population during these trials. However, the results of studies have shown that the hospitalization rate of baloxavir was lower than that of oseltamivir, and to obtain more conservative results, we conservatively estimated the hospitalization rate for baloxavir to be 72.7% of that observed with oseltamivir (Takazono et al., 2024; Neuberger et al., 2022; Komeda et al., 2021).

The ICU admission rates and hospitalization data were extracted from real-world data encompassing 1,800 patients across 111 hospitals in China. Given the variability in hospitalization rates across different hospital levels, adjustments were made based on the number of patient visits in secondary and tertiary hospitals in China in 2020, which were approximately 1.8 and 1.16 billion, respectively (National Health Commission of the People’s Republic of China, 2021).

According to the literature, the duration of full recovery from influenza was 7.425 days; by setting the duration of illness for outpatients with influenza to 1 day, the duration of recovery was 6.425 days. Similarly, the duration of influenza complications was assumed to be 1 day, and the recovery period was set at 7 days (Kamal et al., 2017). In addition, patients who did not receive treatment had a longer recovery period of approximately 8.825 days (NICE. NICE TA168 - Amantadine, 2009). The duration of illness for inpatient data was based on the real-world data. The duration of recovery for inpatient data was calculated using the recovery time for each complication from the published literature, weighted by the proportion of patients with each complication of influenza in real-world data. The specific clinical data are shown in Table 1.

**TABLE 1:** Key input parameters.

Variable	High risk			Healthy		
	Value	Distribution	Source	Value	Distribution	Source
Complications (baloxavir)	0.0210	—	Shionogi (2018a)	0.0430	—	Shionogi (2018b)
Complications (oseltamivir)	0.0360	—	Shionogi (2018a)	0.0210	—	Shionogi (2018b)
ICU fatality rate (baloxavir)	0	—	Shionogi (2018a)	0	—	Shionogi (2018b)
ICU fatality rate (oseltamivir)	0.003	—	Shionogi (2018a)	0	—	Shionogi (2018b)
Clinical data						
% Complication with oseltamivir						
Pneumonia	0.1429	—	Shionogi (2018a)	0.1250	—	Shionogi (2018b)
Bronchitis	0.6429	—	Shionogi (2018a)	0.7500	—	Shionogi (2018b)
Otitis media	0.0714	—	Shionogi (2018a)	0.1250	—	Shionogi (2018b)
Nasosinusitis	0.1429	—	Shionogi (2018a)	0.0000	—	Shionogi (2018b)
% ICU in inpatient	0.0514	—	^b^	0.0102	—	^b^
% Inpatient	0.2353	—	^b^	0.2167	—	^b^
% Complication with baloxavir						
Pneumonia	0.0000	—	Shionogi (2018a)	0.1250	—	Shionogi (2018b)
Bronchitis	0.8750	—	Shionogi (2018a)	0.5625	—	Shionogi (2018b)
Otitis media	0.0000	—	Shionogi (2018a)	0.1250	—	Shionogi (2018b)
Nasosinusitis	0.1250	—	Shionogi (2018a)	0.1875	—	Shionogi (2018b)
Duration of illness (days)						
Pneumonia	1	γ	*	1	γ	*
Bronchitis	1	γ	*	1	γ	*
Otitis media	1	γ	*	1	γ	*
Nasosinusitis	1	γ	*	1	γ	*
Inpatient	6.4803	γ	^a^	4.3283	γ	^a^
ICU	5.5000	γ	^a^	4.0000	γ	^a^
Duration of recovery (days)						
Influenza	6.425	γ	Jiang et al. (2022)	6.425	γ	Jiang et al. (2022)
Pneumonia	7	γ	*	7	γ	*
Bronchitis	7	γ	*	7	γ	*
Otitis media	7	γ	*	7	γ	*
Nasosinusitis	7	γ	*	7	γ	*
Non-treatment	8.825	γ	*	8.825	γ	*
Inpatient	7.5309	γ	Jiang et al. (2022) ^a^	6.9686	γ	Jiang et al. (2022) ^a^
ICU	9.8267	γ	Jiang et al. (2022) ^a^	11.715	γ	Jiang et al. (2022) ^a^
Cost data ($)						
Cost data with oseltamivir						
Influenza	15.36	γ	^c^	15.93	γ	^c^
Pneumonia	40.52	γ	^c^	40.19	γ	^c^
Bronchitis	24.35	γ	^c^	43.54	γ	^c^
Otitis media	14.59	γ	^c^	20.92	γ	^c^
Sinusitis^*^	10.04	γ	^c^	10.04	γ	^c#^
Inpatient	801.03	γ	^c^	426.51	γ	^c^
ICU	5113.39	γ	^c^	2395.78	γ	^c^
Cost data with baloxavir						
Influenza	44.39	γ	^c^	44.96	γ	^c^
Pneumonia	69.55	γ	^c^	69.22	γ	^c^
Bronchitis	53.38	γ	^c^	72.56	γ	^c^
Otitis media	43.62	γ	^c^	49.95	γ	^c^
Sinusitis^*^	39.07	γ	^c^	39.07	γ	^c#^
Inpatient	830.05	γ	^c^	455.54	γ	^c^
ICU	5142.42	γ	^c^	2424.81	γ	^c^
Utility data (QALYs)						
Health	0.96	β	Jiang et al. (2022)	0.96	β	Jiang et al. (2022)
Influenza	0.81	β	Jiang et al. (2022)	0.81	β	Jiang et al. (2022)
Pneumonia	0.63	β	Jiang et al. (2022)	0.63	β	Jiang et al. (2022)
Bronchitis	0.66	β	Shionogi (2018a)	0.66	β	Shionogi (2018a)
Otitis media	0.66	β	Shionogi (2018a)	0.66	β	Shionogi (2018a)
Nasosinusitis	0.66	β	Shionogi (2018a)	0.66	β	Shionogi (2018a)
ICU	0.48	—	Jiang et al. (2022), Shionogi (2018a) ^w^	0.46	—	Jiang et al. (2022), Shionogi (2018a) ^w^
Post-ICU	0.90	β	Shionogi (2018a)	0.90	β	Shionogi (2018a)
Inpatient	0.67	—	Jiang et al. (2022), Shionogi (2018a) ^w^	0.77	—	Jiang et al. (2022), Shionogi (2018a) ^w^

^a^From real-world data. ^b^Real-world data were adjusted based on the number of visits to secondary and tertiary hospitals in China in 2020. ^c^Real-world data were adjusted based on the expenses in secondary and tertiary hospitals in China in 2020. *Estimated duration of illness and recovery. ^w^The QALY values for inpatient and ICU status were weighted based on real-world data on different complication proportions.

^#^The cost data of patients with sinusitis in otherwise healthy population were not available, and the data of the high-risk population were used instead.

#### 2.4.2 Cost data

The cost data were derived from real-world sources. Due to the small and unrepresentative number of high-risk patients in the ICU of tertiary hospitals, we estimated their costs indirectly. This estimation was based on the actual costs of high-risk patients in the ICU of secondary hospitals, which was then adjusted using the national ratio of per capita inpatient costs between tertiary and secondary hospitals in China (2020) (National Health Commission of the People’s Republic of China, 2021). The reported per capita costs were $2,093.772 for tertiary hospitals and $980.124 for secondary hospitals, yielding a cost ratio of approximately 2.14. This ratio was applied to scale the ICU costs from secondary to tertiary hospitals. Additionally, the cost data comprise two components: antiviral drug costs and other treatment costs (Table 1), with no discount applied, owing to the 1-year time horizon of the analysis (Skrzeczek et al., 2021).

#### 2.4.3 Utility data

The utility of hospitalized patients was combined with real-world data, weighted by the number of patients with different complications. Additionally, the utility of ICU patients is lower than that of healthy individuals. Considering premature death, with the median age of patients in the phase III clinical trial of baloxavir being 53 years and the average life expectancy in China being 77.4 years, it was calculated that each death results in a loss of 13.32 QALYs. The utility data for the other states are shown in Table 1.

#### 2.4.4 The parameters of the transmission dynamics model

A dynamic model based on the susceptible exposed infected recovered (SEIR) model was developed to simulate the impacts of antiviral drugs on reducing transmission (Jiang et al., 2022). The parameters in the transmission model included the proportion of natural immunity, vaccine usage, antiviral drug usage, and drug resistance among infected viruses. The transmission dynamics model indicated that baloxavir reduces the incidence of influenza compared with oseltamivir: 3,195 influenza infections per 10,000 people in the high-risk population with baloxavir and 3,567 with oseltamivir. In the otherwise healthy population, there were 2,780 infections per 10,000 people and 3,428 with baloxavir and oseltamivir.

All analyses were conducted using Treeage Pro 2019.

### 2.5 Sensitivity analysis

Sensitivity analyses were conducted to assess the impact of the model input variables on the results and to verify the robustness of the model. The first was one-way sensitivity analysis, particularly adjusting each parameter by ± 20% (Bae et al., 2022) independently of the base value. In addition, the probabilistic sensitivity analysis (PSA, 1000 simulations) was performed to assess the overall sensitivity of the model results.

## 3 Results

### 3.1 Cost-effectiveness results

The results of the base case analysis are shown in Table 2. Baloxavir was dominant in the high-risk population and the otherwise healthy population. In the high-risk population, the total expenditure per patient was $7.7994, with a QALY gain of 0.95885 for the strategy with baloxavir. In addition, the total expenditure per patient was $10.3462 with a QALY gain of 0.95867 for the strategy with oseltamivir; that is, compared to oseltamivir, baloxavir treatment would save $2.5468 per patient and gain an additional 0.00018 QALYs. For the otherwise healthy population, the total cost of baloxavir was $3.9703 with a QALY gain of 0.95903, whereas the total cost of oseltamivir was $5.0981 with a QALY gain of 0.95879. Thus, the otherwise healthy population would save $1.1278 and gain an additional 0.00024 QALYs by choosing baloxavir to treat seasonal influenza.

**TABLE 2:** Base case analysis results.

Populations	Treatments	Cost ($)	Inc. cost ($)	QALYs	Inc. QALY	ICER $/QALY
High- risk	Bal	7.7994	0	0.95885	0	(Dominated)
	Ose	10.3462	2.5468	0.95867	−0.00018	−14396.56596
Healthy	Bal	3.9703	0	0.95903	0	(Dominated)
	Ose	5.0981	1.1278	0.95879	−0.00024	−4692.917286

### 3.2 Sensitivity analysis

**FIGURE 2:**
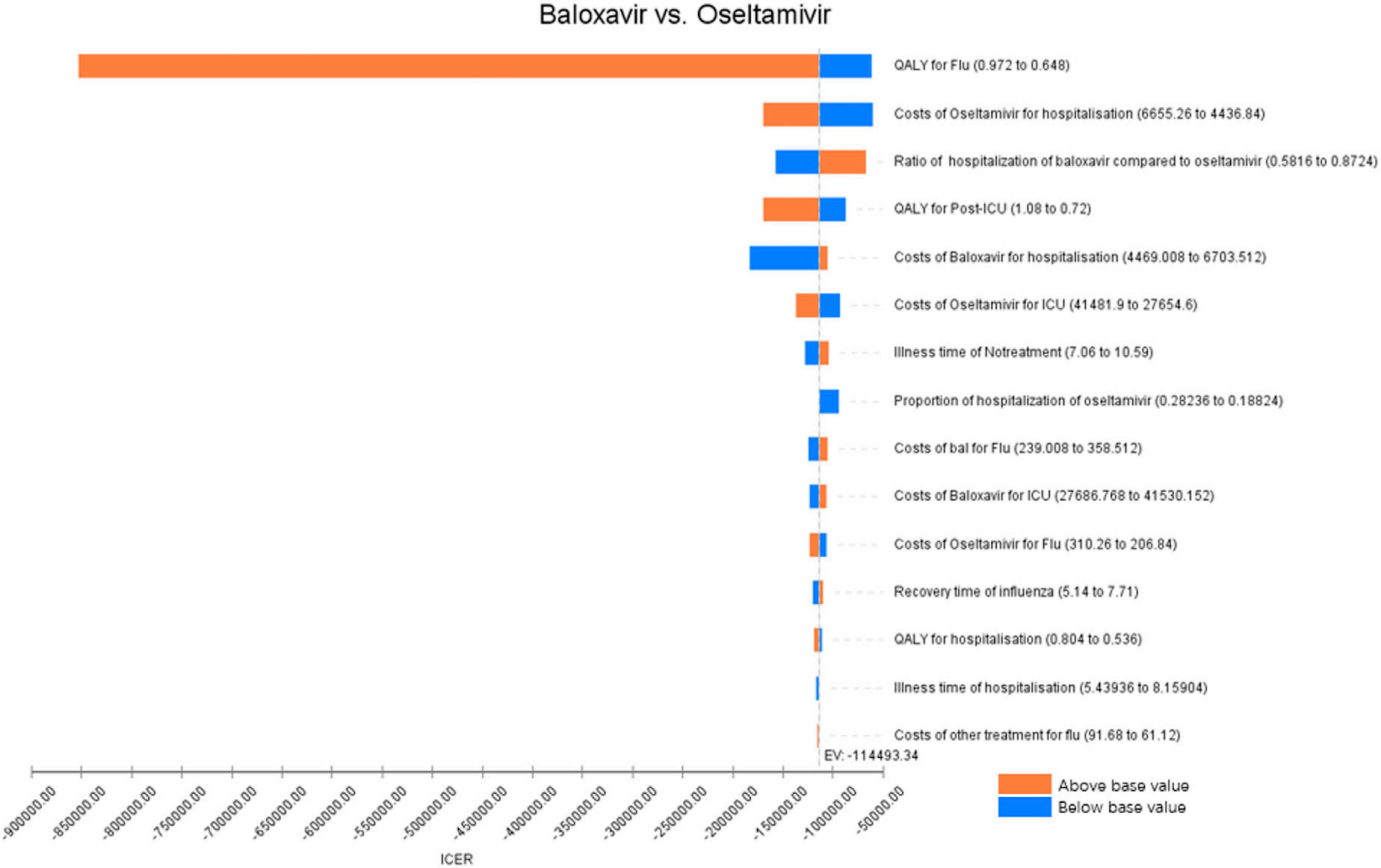
One-way sensitivity analysis (high-risk population).

**FIGURE 3:**
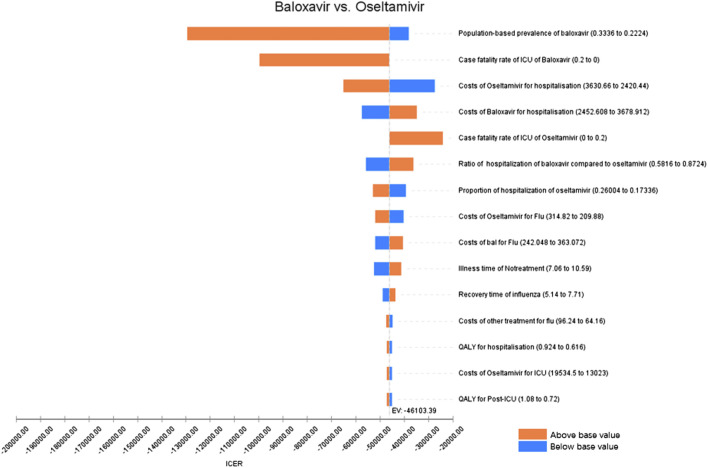
One-way sensitivity analysis (otherwise healthy population).

First, the results of one-way sensitivity analyses were presented using the Tornado diagram (Figures 2, 3), which showed that the main factors affecting the results differed between the high-risk population and the otherwise healthy population. For the high-risk population, the main factors were QALYs for influenza, hospitalization costs, and the ratio of hospitalization of baloxavir to oseltamivir, whereas for the otherwise healthy population, the number of infections with baloxavir, ICU fatality rate, and hospitalization costs were the main factors.

**FIGURE 4:**
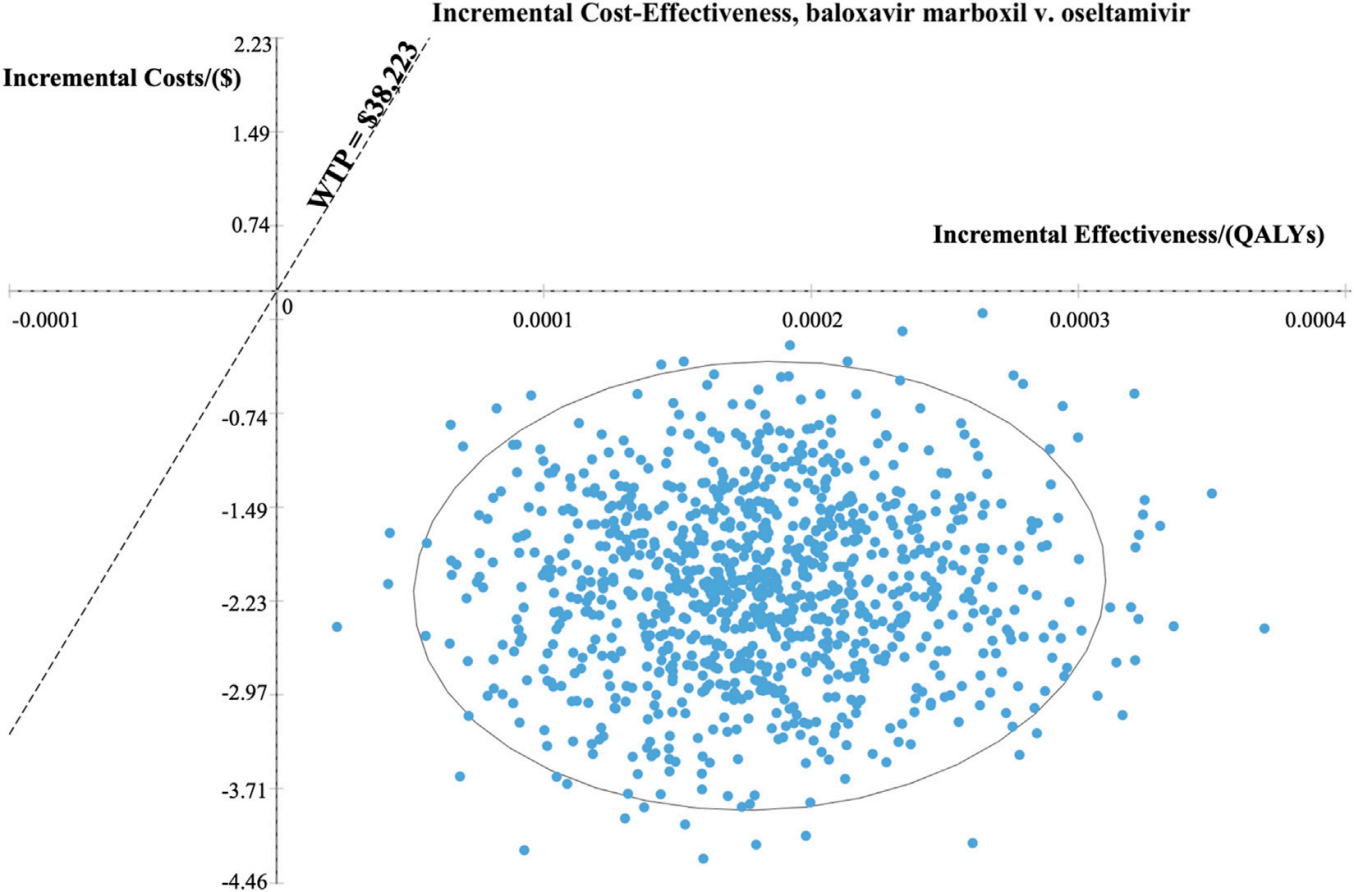
Probability sensitivity analysis at three times GDP (high-risk population).

**FIGURE 5:**
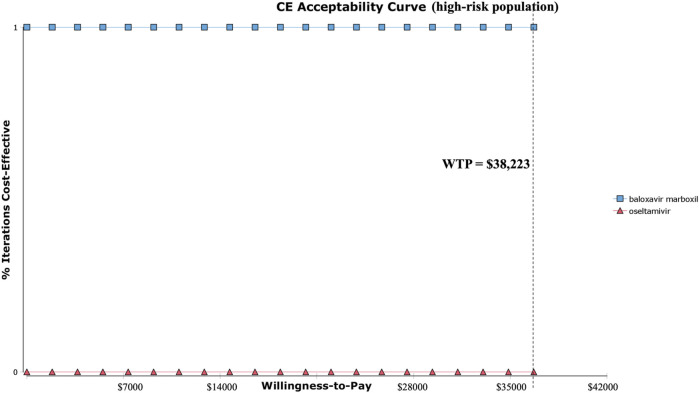
Cost-effectiveness acceptability curve (high-risk population).

At the WTP threshold of $38,223.34 per QALY (three times China’s 2022 GDP *per capita*), baloxavir had a 100% probability of being cost-effective compared to oseltamivir in both the high-risk and otherwise healthy populations. Figures 4, 5 present the results of the PSA and the cost-effectiveness acceptability curve (CEAC) for the high-risk population, respectively. The PSA, based on 1,000 simulations, showed that compared with oseltamivir, baloxavir yielded incremental QALYs mainly ranging from 0.0001 to 0.0004. The costs for baloxavir were consistently lower than those for oseltamivir, with the incremental cost remaining negative throughout. Furthermore, the cost-effectiveness acceptability curve in Figure 5 demonstrated that baloxavir was a dominant strategy.

**FIGURE 6:**
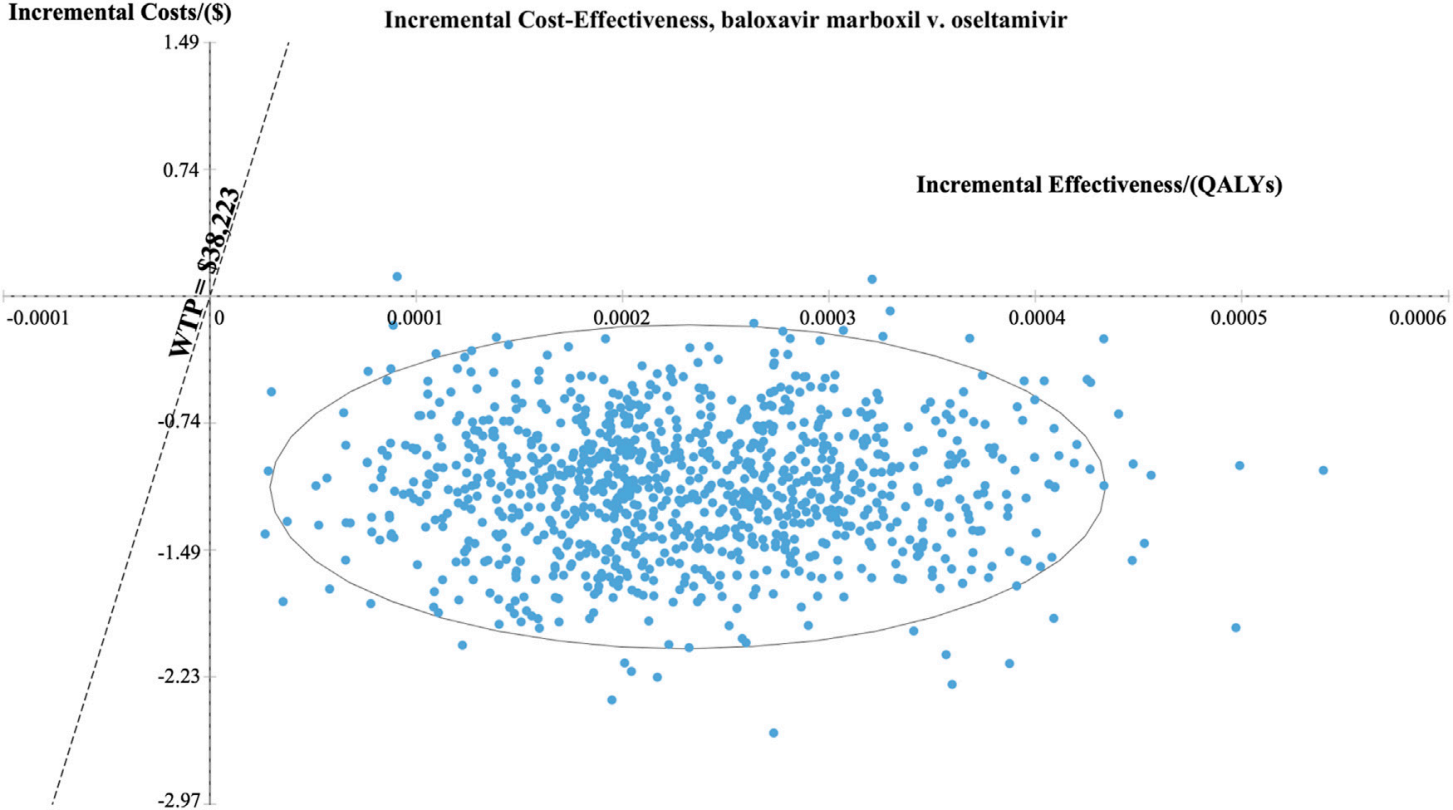
Probability sensitivity analysis at three times GDP (otherwise healthy population).

**FIGURE 7:**
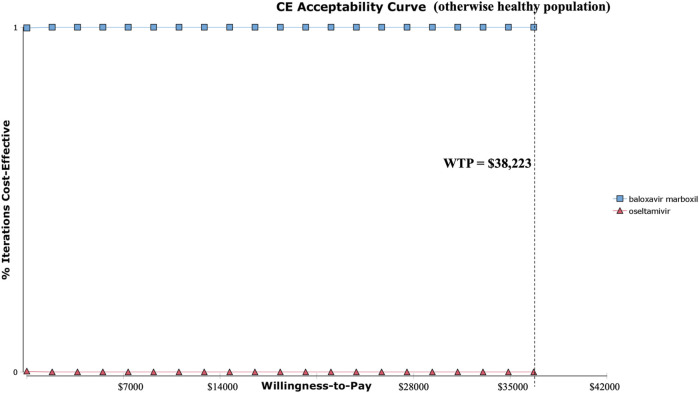
Cost-effectiveness acceptability curve (otherwise healthy population).

Similarly, Figures 6, 7 show the PSA and CEAC results for the otherwise healthy population. The PSA indicated that the incremental QALYs for baloxavir compared to oseltamivir were primarily distributed between 0.0000 and 0.0006. The costs were mostly lower than those of oseltamivir, with only a few rare instances where the costs were slightly higher than oseltamivir, but this did not affect the conclusion and robustness. As illustrated by the cost-effectiveness acceptability curve in Figure 7, baloxavir remained a dominant strategy.

## 4 Discussion

In this study, we demonstrate that from the Chinese healthcare system perspective, baloxavir represents a dominant strategy compared to oseltamivir for both high-risk and otherwise healthy populations, providing greater health benefits and reducing healthcare costs. This outcome is likely driven by three factors. First, baloxavir reduces hospitalization rates (Takazono et al., 2024) and the incidence of complications (Manuel et al., 2024) compared to oseltamivir, directly avoiding subsequent medical expenses. Second, by accelerating viral clearance, it rapidly alleviates symptoms and shortens the course of illness (Hayden et al., 2022), which may decrease the need for follow-up visits. Furthermore, baloxavir can reduce influenza transmission within populations (Hayden et al., 2022), thereby preventing secondary cases and their associated costs.

Sensitivity analysis further reveals population heterogeneity in the key drivers of baloxavir’s cost-effectiveness. For the high-risk population, the ICER is most influenced by influenza-related utility (QALYs), hospitalization costs, and the hospitalization rate ratio between baloxavir and oseltamivir. This can be attributed to the fact that high-risk individuals typically experience longer symptom duration and more severe health utility impairment following influenza infection (Near et al., 2022; de Courville et al., 2022). By substantially shortening symptom duration and accelerating clinical recovery (Hayden et al., 2022), baloxavir effectively improves quality of life in this group. Moreover, given their higher risk of complications and hospitalization (Near et al., 2022), baloxavir can reduce hospitalization rates, thereby avoiding associated high medical costs. This is also a primary source of its cost-effectiveness. Moreover, for the otherwise healthy population, the ICER is primarily driven by the number of infections, ICU mortality, and hospitalization costs. This pattern suggests that baloxavir in this group may depend more on its public health benefit in reducing transmission. Clinical evidence confirms that baloxavir rapidly lowers viral load, thereby decreasing influenza spread within households and communities (Hayden et al., 2022; Takazono et al., 2024; Best et al., 2024). These transmission-blocking effects help curb secondary infections, indirectly enhancing its cost-effectiveness. Although hospitalization rates are generally low in the healthy population, medical costs related to severe outcomes remain substantial. Therefore, by reducing both infection incidence and hospitalization expenses, baloxavir demonstrates an important economic value in this population as well.

Methodologically, in this study, we used modeling to improve the comprehensiveness and local relevance of the evaluation. First, we constructed an evaluation model that integrates transmission dynamics. Compared to static evaluation models, it can capture the public health value derived from baloxavir’s ability to suppress influenza transmission. As shown in a U.S. study by Kommandantvold SA et al., the conclusion may change when influenza transmission is considered, demonstrating that baloxavir becomes a dominant strategy compared to oseltamivir once it reduces the transmission rate by more than 12% (Kommandantvold et al., 2024a). This suggests that neglecting transmission effects may systematically underestimate the overall value of baloxavir. Second, regarding cost parameters, we utilized real-world data derived from the Chinese healthcare setting, thereby ensuring the local applicability of the study findings.

Based on the localized model, we compared our findings within an international context. Although this study aligns with economic evaluation research from Japan, the United States, the United Kingdom, and other countries in supporting that baloxavir reduces utility loss compared to oseltamivir or zanamivir (Skrzeczek et al., 2021; Dronova et al., 2021; Kommandantvold et al., 2024a; Kommandantvold et al., 2024b; van der Pol et al., 2024), consistent results were not obtained regarding cost outcomes. Our findings indicate that baloxavir represents a dominant choice over oseltamivir in both high-risk and otherwise healthy populations, offering higher utility at lower cost. Conversely, studies in Japan, the United States, the United Kingdom, and New Zealand suggest that while baloxavir improves utility, it also incurs higher costs (Skrzeczek et al., 2021; Dronova et al., 2021; Kommandantvold et al., 2024a; Kommandantvold et al., 2024b; van der Pol et al., 2024). By comparing these studies, we speculate that cost-effectiveness disparities likely stem from variations in healthcare costs and drug expenses across countries. In particular, China’s healthcare costs for influenza-related outpatient visits, hospitalizations, and ICUs are generally lower than those in developed countries. For instance, outpatient fees in China typically do not exceed $30, whereas they are approximately $170 in Japan, approximately $250 in the UK, approximately $800 in New Zealand, and range from $1,000 to several thousand dollars in the US, along with cases involving severe complications exceeding $12,000. For hospitalizations, China’s inpatient costs range from $400 to $800, compared to $700 in the UK, $1,500 to $8,000 in Japan, approximately $8,000 in New Zealand, and $10,000 to $30,000 in the US. ICU costs also show significant variation: China charges $2,400–5,100, the UK ranges from $4,500 to $55,000, whereas the US typically costs $60,000, with ICU expenses for otitis media reaching as high as $250,000. On the one hand, healthcare costs may be influenced by economic development levels. On the other hand, it may also relate to differences in diagnostic and treatment practices across countries. In China, physicians often manage influenza empirically based on experience, relying less on laboratory testing, which contributes to lower healthcare costs than those of other nations. Additionally, drug costs play an important role in the final cost outcome. Due to regional pricing for baloxavir, drug prices vary slightly across countries. Studies in other nations have shown that the higher drug costs of baloxavir offset savings from reduced complications and adverse events, resulting in overall high costs (Skrzeczek et al., 2021; Dronova et al., 2021). However, at the time of this study, baloxavir had been launched in China and formally included in the 2021 National Reimbursement Drug List under List B (Mabaroxavir, 2021), which would further impact the drug’s cost and cost-effectiveness in China.

Following the discussion on cost parameters, we further evaluated whether variations in key clinical parameters would impact the conclusions of this study. We noted discrepancies in certain epidemiological parameters between this study and other countries, notably the probability of complications. This parameter in our study was derived from phase III clinical trial results (CAPSTONE) (Shionogi, 2018a; Shionogi, 2018b), showing rates of 2.10% and 3.60% in the baloxavir and oseltamivir groups among high-risk populations, respectively, and 4.30% and 2.10% among the otherwise healthy population. In contrast, a U.S. real-world study based on administrative claims data (Kommandantvold et al., 2024a) reported substantially higher rates: 30.5% and 31.3% for high-risk populations, and 26.2% and 28.1% for the healthy population. We speculate that the discrepancy primarily stems from differences between clinical trials and clinical practice. The CAPSTONE trials, as strictly controlled clinical studies, enrolled carefully selected patients with confirmed influenza. In comparison, the U.S. real-world study likely included a broader patient population with more comorbidities or applied less restrictive diagnostic criteria. We argue that, despite this difference in the absolute parameter value, its impact on the study’s conclusions is likely limited. First, the results of the sensitivity analyses demonstrate that the economic advantage of baloxavir over oseltamivir remains stable within a certain range of complication probability fluctuations. Furthermore, within the analytic framework of the model, the results of an economic evaluation primarily depend on the incremental costs and health outcomes associated with the interventions. The absolute probability of complications influences the total costs for both treatment strategies in parallel, whereas the relative risk difference between the two strategies is likely a more critical driver of the incremental results. Therefore, although the U.S. real-world data report higher absolute probabilities, the relative risk difference between the baloxavir and oseltamivir groups is small. Consequently, this discrepancy does not undermine the robustness of our primary conclusion.

This study still has some limitations. First, the epidemiological parameters primarily derive from the results of the phase III clinical trial (CAPSTONE) (Shionogi, 2018a; Shionogi, 2018b), a data source commonly selected for similar international economic evaluations (Skrzeczek et al., 2021; Dronova et al., 2021; van der Pol et al., 2024). However, although the CAPSTONE trial sites were widely distributed across Japan, the United States, Canada, Europe, South Korea, and other countries and regions, it did not include research centers in mainland China. Patient healthcare-seeking behaviors, population immunity backgrounds, and clinical diagnostic criteria for complications may vary across countries or regions. Therefore, directly applying data from this international clinical trial to the Chinese context has certain limitations. Furthermore, the stringent diagnostic criteria used in clinical trials may result in reported complication rates underestimating the actual incidence rates in real-world Chinese healthcare settings. Although sensitivity analysis results indicate that these factors do not compromise the robustness of the conclusions, incorporating efficacy data from the Chinese context in future studies would further enhance the accuracy of the assessment. Second, the cost data in this study are based on influenza-like cases treated with antiviral drugs. As clinical practice relies primarily on symptoms rather than laboratory confirmation for diagnosing influenza-like cases, the inclusion criteria may have encompassed some patients with other acute respiratory diseases in addition to influenza. It may result in cost data not entirely attributable to influenza itself, potentially leading to an overestimation of the per capita medical costs for influenza. Finally, as with other studies, when handling cost parameters, the cost data for both baloxavir and oseltamivir regimens differed only in drug expenses, with no distinction in other clinical costs. This may lead to an overestimation of baloxavir’s costs, potentially introducing some inaccuracies in the results. However, this does not fundamentally alter the study’s conclusion, as baloxavir remains less costly than oseltamivir even when its costs are overestimated. Additionally, at the time of the study, the approved indications for baloxavir did not include the pediatric population under 12 years of age. However, children are one of the susceptible populations for influenza, and they are more likely to be a source of transmission of influenza viruses at home and in school. The lack of exploration of this population may underestimate the actual economic viability of the drug, and therefore, further studies could be conducted.

## Data Availability

The original contributions presented in the study are included in the article/supplementary material, further inquiries can be directed to the corresponding author.
